# Effect of levodopa‐carbidopa intestinal gel on dyskinesia in advanced Parkinson's disease patients

**DOI:** 10.1002/mds.26528

**Published:** 2016-01-28

**Authors:** Angelo Antonini, Victor S. C. Fung, James T. Boyd, John T. Slevin, Coleen Hall, Krai Chatamra, Susan Eaton, Janet A. Benesh

**Affiliations:** ^1^ Parkinson and Movement Disorders UnitIRCCS Hospital San CamilloVeniceItaly; ^2^ Movement Disorders UnitWestmead Hospital and Sydney Medical SchoolSydneyAustralia; ^3^ Department of NeurologyUniversity of Vermont College of MedicineBurlingtonVermontUSA; ^4^ University of Kentucky College of MedicineLexingtonKentuckyUSA; ^5^ AbbVie, Inc.North ChicagoIllinoisUSA

**Keywords:** Parkinson's disease, levodopa‐carbidopa intestinal gel, infusion, dyskinesia, percutaneous endoscopic gastrojejunostomy, carbidopa‐levodopa enteral suspension

## Abstract

**Objective:**

The purpose of this study was to assess the effect of levodopa‐carbidopa intestinal gel (carbidopa‐levodopa enteral suspension) in advanced Parkinson's disease patients with troublesome dyskinesia.

**Methods:**

Post hoc analyses of patient data from a 12‐week, randomized, double‐blind study and a 54‐week open‐label study were performed. Efficacy was assessed in the subgroup of patients defined by ≥1 hour of “on” time with troublesome dyskinesia at baseline as recorded in Parkinson's disease symptom diaries (double blind: n = 11 levodopa‐carbidopa intestinal gel, n = 12 oral levodopa‐carbidopa; open label: n = 144 levodopa‐carbidopa intestinal gel). The changes in “off” time, “on” time with and without troublesome dyskinesia, and the overall safety and tolerability of levodopa‐carbidopa intestinal gel were analyzed.

**Results:**

Although not significantly different from oral levodopa treatment (*P* > .05) in the double‐blind study, levodopa‐carbidopa intestinal gel treatment resulted in a reduction from baseline in “on” time with troublesome dyskinesia (mean [standard deviation] hours: baseline = 3.1 [1.7], change from baseline to final = −1.8 [1.8], *P* = .014), increase in “on” time without troublesome dyskinesia (baseline = 7.4 [2.2], change = 4.4 [3.6], *P* = .004), and decrease in “off” time (baseline = 5.5 [1.3], change = −2.7 [2.8], *P* = .015). Similar trends were found in the open‐label study. An increase in levodopa‐carbidopa intestinal gel dose was not significantly correlated with increased “on” time with troublesome dyskinesia in either study (double blind: *r* = −.073, *P* = .842; open label: *r* = −0.001, *P* = .992). Adverse events were usually mild to moderate in severity and related to the gastrointestinal procedure.

**Conclusion:**

Our exploratory analyses suggest that optimizing levodopa delivery with levodopa‐carbidopa intestinal gel may reduce troublesome dyskinesia in advanced Parkinson's disease. © 2016 International Parkinson and Movement Disorder Society

## Introduction

Parkinson's disease (PD) is a common neurodegenerative disorder^1^ characterized by a progressive depletion of dopaminergic neurons in the nigrostriatal system^2^ and motor dysfunctions such as bradykinesia, rigidity, tremor, and postural instability.^1^ Immediate release oral levodopa‐carbidopa (LC‐IR) is the leading treatment for PD.^3^, ^4^ However, over time, LC‐IR‐treated PD patients develop complications of motor fluctuations and dyskinesia that are related in part to pulsatile stimulation and erratic gastric emptying.^5^, ^6^, ^7^ These complications are often difficult to treat and negatively impact quality of life.^8^ In particular, dyskinesia has a negative effect on measures of cognition, activities of daily living, social stigma, and bodily discomfort.^8^ Currently there are no medications approved to treat dyskinesia, although some benefit has been shown with amantadine and continuous subcutaneous apomorphine.^9^, ^10^ Deep brain stimulation of the subthalamic nucleus and globus pallidus internus is efficacious, but there are significant restrictions as a result of age and cognitive behavioral state, which limit its clinical application.^11^

Levodopa‐carbidopa intestinal gel (LCIG) is a treatment option for advanced PD patients. LCIG is administered to the upper intestine via a percutaneous endoscopic gastrojejunostomy (PEG‐J) tube and portable infusion pump. Previously published data demonstrated that switching from oral levodopa to LCIG significantly reduced “off” time,^12^ with the benefits sustained for at least 12 months.^13^, ^14^ In this study, we performed a post hoc analysis of the efficacy of LCIG in a subgroup of patients with ≥ 1 hour of “on” time with troublesome dyskinesia (TSD) at baseline who were enrolled in a double‐blind study, and we sought to confirm the findings using post hoc analyses from a larger, open‐label study.

## Methods

### Study Designs

Data from two previously described studies^12^, ^13^ were used in this analysis. The study protocols for each study were approved by every participating institution's internal review board or ethics committee. All patients provided written informed consent. LCIG (designated in the United States as carbidopa‐levodopa enteral suspension) was continuously infused during waking hours (approximately 16 hours). The total daily dose of LCIG per day was composed of 3 individually adjusted doses (morning, continuous maintenance, and extra). All patients were optimized to LC‐IR prior to baseline measurements.

The primary study (NCT00357994/NCT00660387) was a 12‐week, randomized, double‐blind, double‐dummy, parallel‐group, phase 3 study.^12^ All 71 enrolled patients received a PEG‐J and were randomized to either LCIG (n = 37) or LC‐IR (n = 34). The LCIG cohort received placebo tablets identical in size and color to LC‐IR, and the placebo cohort received placebo gel. Patients were allowed to continue stable regimens of anti‐PD medications with the exception of apomorphine. PD symptom diaries (see Efficacy section) were assessed at baseline and weeks 2, 3, 4, 6, 8, 10, and 12.

The second study (NCT00335153) was a 54‐week, open‐label, multicenter, phase 3 safety study (N = 354) with patients who had PEG‐J placed after titrating to an optimal dose of LCIG via a temporary nasojejunal tube.^13^ Patients were not allowed to continue other anti‐PD medications after the initiation of LCIG infusion and received LCIG monotherapy for at least the first 28 days of treatment. PD symptom diaries were assessed at baseline and weeks 4, 12, 24, 36, and 54.

### Patients

Adults (≥30 years) with advanced PD consistent with UK Brain Bank criteria were enrolled in each study.^12^, ^13^ Patients were eligible if they were levodopa responsive, experienced motor fluctuations despite optimized therapy, and had at least 3 hours of “off” time on baseline diary assessment. Additional qualifying criteria for patients were previously reported.^12^, ^13^

### Efficacy

All patients with a baseline efficacy measurement as well as at least 1 postbaseline efficacy measurement were included in the analysis of efficacy. Patients recorded their motor status in 30‐minute intervals as either “off” time, “on” time without dyskinesia, “on” time with non‐TSD, “on” time with TSD, or asleep at home using the PD symptom diary.^15^ The efficacy endpoints in each study included the mean change from baseline in the number of hours of “off” time, “on” time without TSD (dyskinesia and non‐TSD), and “on” time with TSD as recorded in the PD symptom diary and the mean change from baseline to the final visit on the scores from the UPDRS dyskinesia questions (UPDRS part IV, questions 32‐34 regarding dyskinesia).

### Safety

All patients who received LCIG treatment were included in the analyses of safety. Evaluation of the safety and tolerability of LCIG included adverse event (AE) monitoring. AEs were coded using the Medical Dictionary for Regulatory Activities (MedDRA) version 14.0, and tabulated by MedDRA Preferred Term. Each event could be coded to more than one relevant preferred term. All AEs presented are treatment‐emergent AEs, defined as those with an onset on or after the initial PEG‐J (double‐blind) or nasojejunal (open‐label) tube placement and no more than 30 days after PEG‐J removal. Study investigators rated each event as mild, moderate, or severe.

### Troublesome Dyskinesia Subgroup and Statistical Analysis

Unless noted otherwise, all analyses reported were performed with a subgroup of patients with TSD, defined post hoc as those with an average of at least 1 hour of TSD per day during the 3 consecutive days prior to baseline (after optimization to LC‐IR but prior to start of study drug) as recorded in the PD symptom diary. Data from the TSD subgroup were analyzed separately from each study, and no corrections were made for multiple testing in these post hoc analyses.

Values from the PD symptom diaries were normalized to a 16‐hour waking day and averaged for the 3 consecutive days prior to each visit. “On” time without TSD was calculated as the sum of “on” time without dyskinesia and “on” time with non‐TSD. However, “on” time without dyskinesia and “on” time with non‐TSD were both included in the assessment of the distribution of time within the 16‐hour waking day.

Baseline was postoptimization with LC‐IR but prior to the start of the study drug. The final value for efficacy measurements was defined as the last nonmissing value assigned to the treatment period for the patient. Within‐group change from baseline to each visit and to endpoint was assessed with a 1‐sample *t* test. A repeated‐measures model with the terms of treatment, country, baseline, and visit and the interaction terms treatment by visit and baseline by visit were used to compare the treatment group diary measures in the double‐blind study. The change from baseline mean daily levodopa dose during the study was assessed with a 1‐sample *t* test. The relationship between the change in daily levodopa dose and the change in “on” time with TSD was examined by scatterplot and Pearson correlation coefficient. AEs and serious AEs were summarized for each study's TSD subgroup.

In addition to the TSD subgroup analysis in each study, a supplemental analysis of the PD symptom diary outcomes was performed on data from subsets of patients in the open‐label study with even higher amounts of TSD at baseline (≥1.5, ≥ 2, ≥ 2.5, and ≥3 hours).

## Results

### Double‐Blind Study

There were 11 (30%) LCIG‐treated patients (n = 37) and 12 (35%) LC‐IR‐treated patients (n = 34) who had at least 1 hour of TSD at baseline. There were no clinically meaningful differences between treatment groups for baseline characteristics (Table 1). In these LCIG‐ and LC‐IR‐treated patients, respectively, the mean (standard deviation [SD]) daily dose of levodopa was 927 (287) mg and 963 (497) mg at baseline, and during the study it was 1019 (310) mg and 1238 (737) mg. “Off” time was significantly reduced for these patients in each treatment group when compared with baseline (mean [SD] change from baseline to final: LCIG, −2.67 [2.83], *P* < .001; LC‐IR, −2.04 [2.80], *P* = .036), and there was no significant difference between treatment groups (*P* = .983).

**Table 1:** Baseline characteristics of patients with troublesome dyskinesia

	Double‐blind study		Open‐label study
Characteristic	LCIG, n = 11	LC‐IR, n = 12	LCIG, n = 144
Age (years), mean (SD)	66.0 (10.2)	65.8 (6.6)	63.4 (9.2)
Sex, n (%)			
Male	7 (63.6)	6 (50.0)	77 (53.5)
Race, n (%)			
White	10 (90.9)	11 (91.7)	134 (93.1)
American Indian or Alaska Native	1 (9.1)	0	0
Asian	0	1 (8.3)	9 (6.3)
Black	0	0	1 (0.7)
PD duration, mean (SD) years	11.6 (5.5)	13.4 (6.4)	13.0 (5.6)
MMSE, mean (SD) score	28.5 (1.3)	29.2 (0.9)	28.4 (1.7)
“On” time with TSD, mean (SD) hours	3.1 (1.6)	3.0 (1.7)	3.4 (1.8)
“On” time without TSD, mean (SD) hours	7.4 (2.1)	6.7 (2.5)	6.8 (2.4)
“Off” time, mean (SD) hours	5.5 (1.3)	6.4 (1.7)	5.8 (2.1)
UPDRS Part IV dyskinesia questions (#32‐34), mean (SD) score	3.5 (1.6)	3.1 (1.6)	5.1 (2.0)^a^
PDQ‐39 summary index, mean (SD) total score	39.4 (19.9)	45.2 (13.4)	43.7 (14.9)
CGI‐S, mean (SD) score	4.5 (0.5)	5.1 (0.7)	4.9 (0.8)^b^

LC‐IR, immediate release oral levodopa‐carbidopa; SD, standard deviation; TSD, troublesome dyskinesia; PDQ‐39, 39‐item Parkinson's Disease Questionnaire; CGI‐S, Clinical Global Impression‐Severity.

a n = 135.

b n = 142.

**Figure 1:**
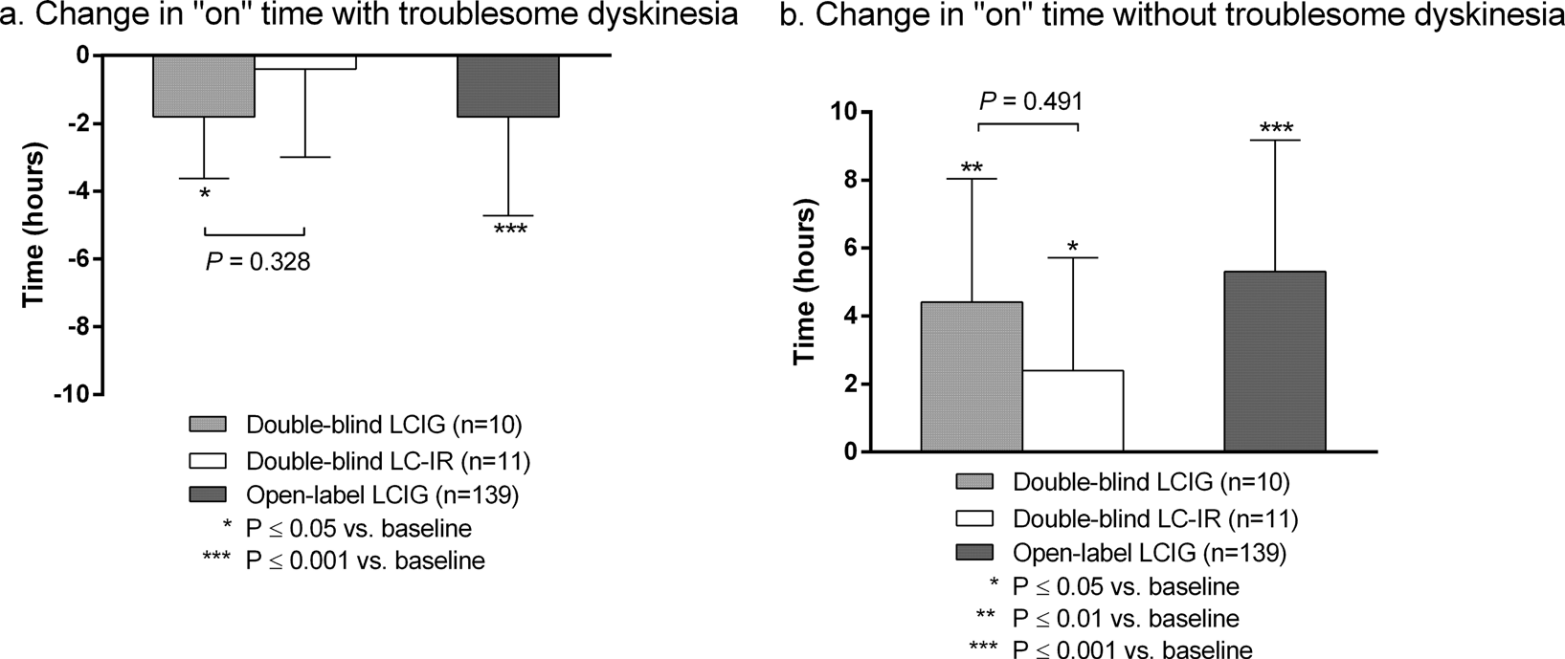
Mean change from baseline to final in daily “on” time. Bars are mean (standard deviation) for daily normalized “on” time measures. *P* values versus baseline are from a 1‐sample *t* test. A repeated‐measures model with the terms of treatment, country, baseline, and visit and the interaction terms treatment by visit and baseline by visit were used to compare treatment groups in the double‐blind study. LCIG, levodopa‐carbidopa intestinal gel; LC‐IR, immediate release levodopa‐carbidopa.

**Figure 2:**
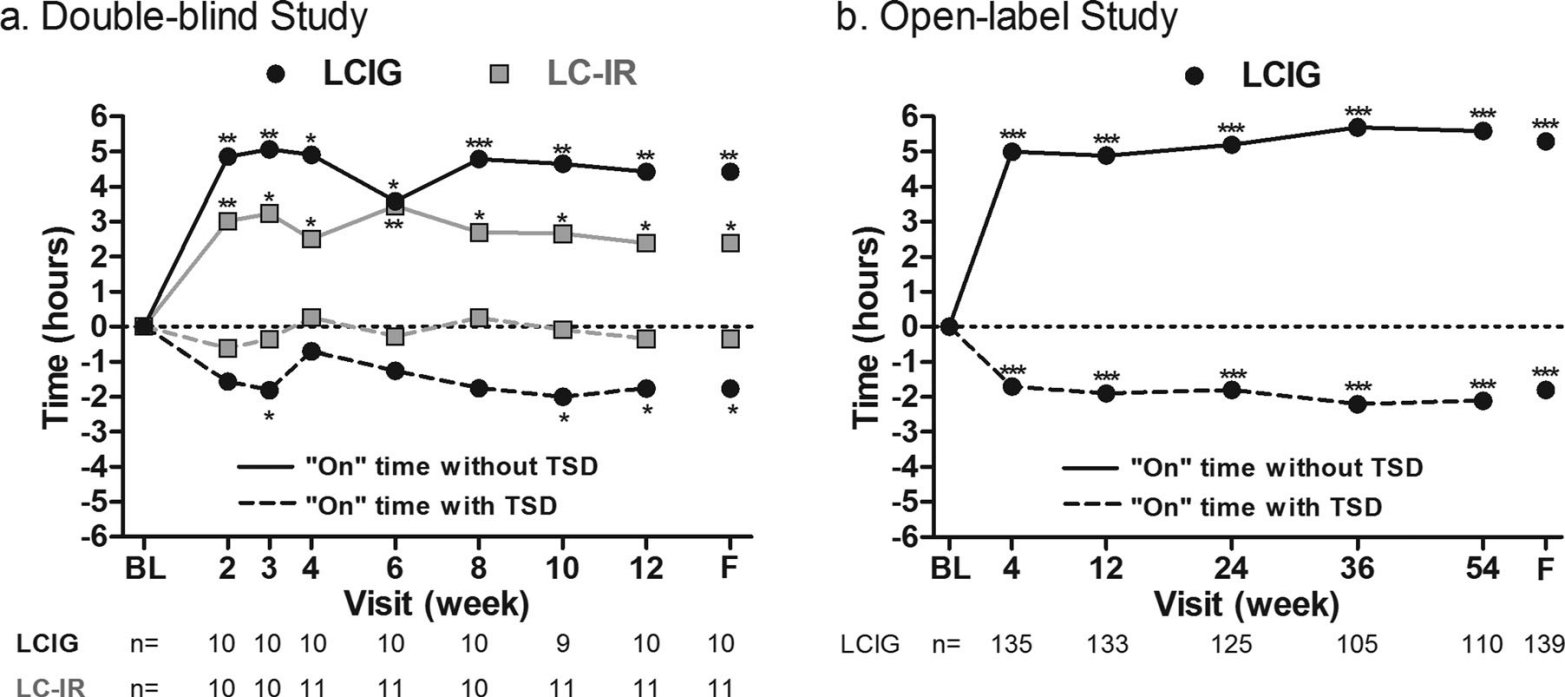
Mean change from baseline over time in daily “on” time. Data shown are mean daily normalized “on” time measures. *P* values (1‐sample *t* test) indicate significance when compared with baseline at ***P ≤ .001,**P ≤ 0.01, and *P ≤ 0.05. LCIG, levodopa‐carbidopa intestinal gel; LC‐IR, immediate release levodopa‐carbidopa; TSD, troublesome dyskinesia; BL, baseline; F, final.

Although not significantly different from LC‐IR (“on” time with TSD, *P* = .328; “on” time without TSD, *P* = .491), LCIG‐treated patients had a significant mean decrease from baseline to final visit in hours of “on” time with TSD (Fig. 1a), which was accompanied by a significant improvement in “on” time without TSD (Fig. 1b). The LC‐IR‐treated patients also reported a significant improvement in “on” time without TSD (Fig. 1b). LCIG‐treated patients showed a trend of improvement in “on” time without TSD and “on” time with TSD from baseline to every time point, starting as early as week 2; however, these improvements were not significant when compared with LC‐IR‐treated patients (Fig. 2a).

**Figure 3:**
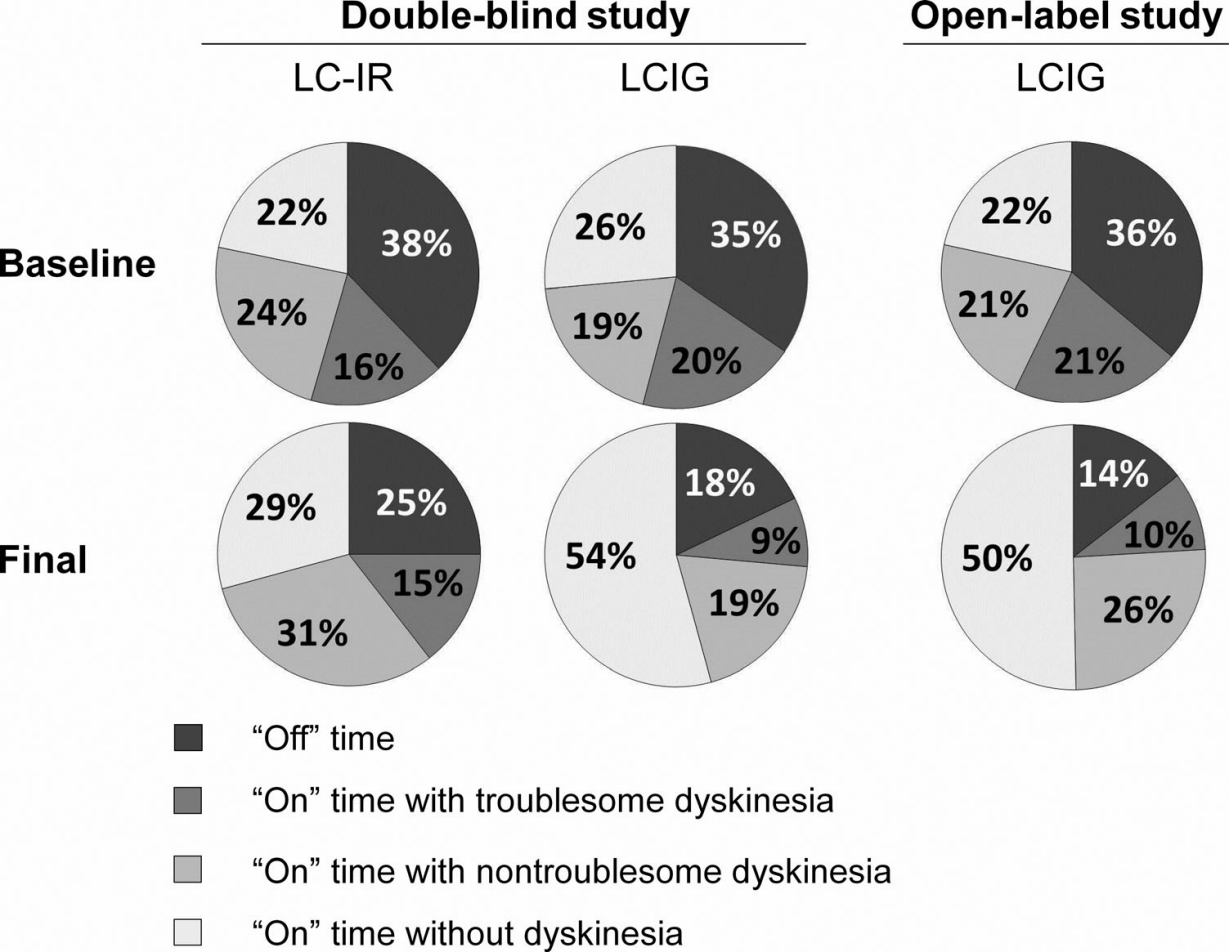
Distribution of time spent in different motor symptoms based on PD diary data. Percentages are of 16 total waking hours. Double‐blind study n = 10 LCIG, 11 LC‐IR; open‐label study n = 139. LCIG, levodopa‐carbidopa intestinal gel; LC‐IR, immediate release levodopa‐carbidopa.

The composition of time spent in the different motor states during the course of the 16‐hour daily treatment period was also examined, further breaking down “on” time without TSD into “on” time without dyskinesia and “on” time with non‐TSD (Fig. 3). For LCIG‐treated patients, the decrease in “off” time at the final visit translated to an increase in “on” time without dyskinesia and a decrease in “on” time with TSD. “On” time with non‐TSD did not change. The decrease in “off” time for LC‐IR‐treated patients was evenly distributed between an increase in “on” time without dyskinesia and “on” time with non‐TSD, whereas the overall “on” time with TSD did not change. The percentage of “on” time without dyskinesia more than doubled in LCIG‐treated patients, whereas it only increased by approximately a third in the LC‐IR patients. An increase in LCIG dose was not significantly correlated with an increase in “on” time with TSD (*r* = −.073, *P* = .842; Supplemental Fig. 1).

LCIG‐treated patients showed no change on the UPDRS Part IV dyskinesia questions (nos. 32‐34) (mean [SD] change from baseline to final = −0.5 [1.57], *P* = .277), whereas LC‐IR patients showed a significant worsening (1.3 [1.37], *P* = .006), though there was no significant difference between treatment groups (*P* > .05).

The overall incidence of AEs and most frequently reported AEs were similar across the treatment groups (Table 2). AEs were usually mild to moderate in severity and related to the gastrointestinal procedure.

**Table 2:** Summary of safety in the double‐blind and open‐label studies

	Double‐blind study, mean (SD)		Open‐label study, mean (SD)
N (%)	LCIG, n = 11	LC‐IR, n = 12	LCIG, n = 144
Any adverse event (AE)	10 (91)	12 (100)	134 (93)
Any serious AE	2 (18)	2 (17)	46 (32)
Discontinued due to an AE	1 (9.1)	0	9 (6.3)
AEs occurring in ≥ 10% of TSD subgroup patients in the open‐label study			
Complication of device insertion^a^	6 (55)	5 (42)	55 (38)
Abdominal pain	5 (45)	3 (25)	44 (31)
Procedural pain	5 (45)	6 (50)	32 (22)
Nausea	5 (45)	1 (8.3)	31 (22)
Insomnia	2 (18)	2 (17)	31 (22)
Excessive granulation tissue	0	0	28 (19)
Constipation	1 (9.1)	2 (17)	24 (17)
Fall	0	3 (25)	23 (16)
Postoperative wound infection	2 (18)	4 (33)	21 (15)
Incision site erythema	1 (9.1)	1 (8.3)	21 (15)
Anxiety	2 (18)	1 (8.3)	19 (13)
Depression	2 (18)	1 (8.3)	18 (13)
Urinary tract infection	0	1 (8.3)	17 (12)
Dyskinesia	2 (18)	2 (17)	16 (11)
Weight decreased	0	0	16 (11)

A single event could be coded to ≥1 preferred term. Terms are from MedDRA version 14.0. SD, standard deviation; LCIG, levodopa‐carbidopa intestinal gel; LC‐IR, immediate release oral levodopa‐carbidopa; TSD, troublesome dyskinesia.

a In the entire study population, events with this term were most often additionally coded to abdominal pain, abdominal discomfort, abdominal distension, flatulence, and pneumoperitoneum.

### Open‐Label Study

Of the 354 patients, 144 (41%) had at least 1 hour of TSD at baseline. Baseline characteristics were similar to both treatment groups in the double‐blind study (Table 1). The mean (SD) daily dose of levodopa in these patients was 1090 (624) mg at baseline and 1489 (500) mg during the study. “Off” time for these patients decreased significantly (mean [SD] change from baseline to final = −3.48 [2.68], *P* < .001).

Similar to the results for the LCIG‐treated patients in the double‐blind study, “on” time with TSD significantly improved from baseline to final visit (Fig. 1a), which was accompanied by a significant improvement in “on” time without TSD (Fig. 1b). In a supplemental analysis, an even greater improvement in “on” time with TSD was observed in patients with higher levels of TSD at baseline; patients with ≥ 3 hours of TSD at baseline (n = 71) reported a mean (SD) decrease of 3.02 (2.98) hours of “on” time with TSD (Supplemental Table 1). An increase in LCIG dose was not significantly correlated with an increase in “on” time with TSD (*r* = −.001, *P* = .992; Supplemental Fig. 1). Patients had a significant improvement in “on” time without TSD and “on” time with TSD from baseline to week 4, which was sustained throughout the 54‐week study (Fig. 2b).

Similar to the results of the double‐blind study, the decrease in “off” time translated to an increase in “on” time without dyskinesia and a decrease in “on” time with TSD at the final visit (Fig. 3). The percentage of “on” time without dyskinesia more than doubled, and “on” time with non‐TSD increased slightly.

Patients showed a significant improvement in the UPDRS Part IV dyskinesia questions (nos. 32‐34; mean [SD] change from baseline to final = −2.2 [2.41], *P* < .001).

The overall incidence of AEs and most frequently reported AEs were similar to those in LCIG‐treated patients in the double‐blind study (Table 2).

## Discussion

Patients with at least 1 hour of “on” time with TSD at baseline were included in this analysis. This subgroup was defined post hoc to allow for a clinically relevant assessment of the effect of LCIG on dyskinesia. Data from double‐blind and open‐label studies were included. The studies differed in design with respect to dose initiation, concomitant antiparkinsonian medication usage, duration of treatment, and comparators, and neither study was powered to detect a change in measures of dyskinesia.

The interpretation of the results is limited because they were exploratory and conducted post hoc. The strength of our findings is that, despite the differences in study designs, the results in the larger sample of patients in the open‐label study confirmed the results in the double‐blind study.

Our analysis of patients with dyskinesia showed that in the double‐blind pivotal study,^12^ patients randomized to LCIG showed significant improvements in the mean “on” time with TSD, mean “on” time without TSD, and mean “off” time; however, these improvements were not significant when compared with LC‐IR. Notably, the decrease from baseline in time spent as “on” time with TSD converted mostly to increased “on” time without dyskinesia during the course of the study because there were minimal changes in “on” time with non‐TSD. When further evaluating patients in the open‐label study with more than 2 hours or 3 hours of baseline TSD, even greater reductions in TSD were observed.

Dyskinesia is difficult to treat in the advanced stage of PD because it becomes more prominent with long‐term oral levodopa replacement therapy.^16^ At this stage in disease, the “on” time of motor fluctuations and dyskinesias have previously been correlated with high levels of levodopa in the plasma or cerebrospinal fluid.^17^, ^18^, ^19^, ^20^ LCIG provides continuous exposure to levodopa during waking hours (16 hours) and has the potential to circumvent complications such as dyskinesia by attenuating peak levodopa levels.^21^ Interestingly, the reduction in TSD was observed after the second week of treatment, and the mean daily levodopa dose during treatment in the double‐blind study was approximately 90 mg higher than baseline and 400 mg higher in the longer, open‐label study (patients in the open‐label trial discontinued other antiparkinsonian therapies, hence the larger increase in levodopa dose). This suggests that an improvement in TSD may be related to the change in levodopa pharmacokinetics and continuous drug delivery. However, peripheral levodopa levels (including C_max_) were not measured in these studies.

Deep brain stimulation (DBS) of the subthalamic nucleus and globus pallidus internus is an alternative option for advanced PD patients, but patient selection is limitated.^11^, ^22^ In a randomized 6‐month study, DBS reduced “on” time with TSD by −2.6 (95% confidence interval = −3.3 to −2.0) hours per day, increased “on” time without TSD by 4.6 (3.8 to 5.3) hours per day, and reduced “off” time by −2.4 (−3.1 to −1.8) hours per day.^23^ Our findings suggest that patients with advanced PD and severe motor fluctuations that include TSD can derive benefits from LCIG of a similar magnitude to DBS, and both therapies should be considered. The data presented here indicate that LCIG may provide a similarly efficacious alternative, and for those patients older than 70 years of age, LCIG may be the only potential option.^22^, ^24^

The safety profile of LCIG treatment in the TSD subgroup was consistent with the safety profile in the total population,^12^, ^13^ with the majority of adverse events associated with the procedure being complication of device insertion, abdominal pain, and procedural pain. In both studies dyskinesia was reported as an AE by only a slightly higher percentage of patients in the TSD subgroup when compared with the entire treatment group: double‐blind LCIG patients = 18% versus 14% (TSD subgroup, entire treatment group), double‐blind LC‐IR patients = 20% versus 12%, and open‐label patients = 11% versus 9%. Overall, the safety data suggest that there are no additional safety concerns for patients with higher baseline TSD that would preclude them from considering LCIG treatment.

Reductions in “off” time are often associated with improved “on” time without TSD. In patients with significant “on” time with TSD, this reduction is associated with improved “on” time without TSD. Therefore, LCIG can effectively reduce “off” time without worsening dyskinesia and decrease TSD without worsening “off” time. The mechanisms by which this effect is accomplished are primarily related to change in levodopa pharmacokinetics. Oral levodopa treatment, even in association with enzyme inhibitors, results in significant oscillations in plasma bioavailability.^25^ This is also aggravated by erratic gastric emptying that makes the levodopa effect difficult to predict in advanced disease.^26^ Our findings suggest that by modifying the pharmacokinetics of levodopa, even with a relative increase in its daily administered dose, changing the mode of its delivery to achieve more constant blood levels may enhance its efficacy, reduce motor response oscillations, and improve troublesome dyskinesia.

## Author Roles

(1) Research Project: A. Conception, B. Organization, C. Execution; (2) Statistical Analysis: A. Design, B. Execution, C. Review and Critique; (3) Manuscript: A. Writing of the First Draft, B. Review and Critique.

A.A.: 1A, 1C, 2C, 3A, 3C

V.C.S.F.: 1C, 2C, 3B

J.T.B.: 1C, 2C, 3B

J.T.S.: 1C, 2C, 3B

C.H.: 2A, 2C, 3B

K.C.: 1A, 1B, 1C, 2A, 2C, 3A, 3B

S.E.: 1B, 1C, 2C, 3B

J.A.B.: 1B, 1C, 2C, 3B

## Financial Disclosures of All Authors for the Past Year

A.A. was a study investigator and has received compensation for consultancy and speaker related activities from UCB, Boston Scientific, Boheringer Ingelheim, AbbVie, Zambon. A.A. received research support from Mundipharma, the Italian Ministry Research grant no. RF‐2009‐1530177, and Horizon 2020 Program grant no. 643706. V.S.C.F. receives a salary from NSW Health, has received research support from the National Health and Medical Research Council of Australia, and is on Advisory Boards and/or has received travel grants from Abbvie Inc., Allergan, Boehringer‐Ingelheim, Hospira, Ipsen, Lundbeck, Novartis, Parkinson's KinetiGraph, Solvay, and UCB. J.T.B. served as a consultant and/or scientific advisor for AbbVie Inc., Auspex, Lundbeck, Medical Education Resources, and Oakstone Medical Publishing and received research support from the Michael J. Fox Foundation, the National Institutes of Health/National Institute of Neurological Disorders and Stroke, Auspex, and Abbvie Inc. J.T.S. has served as an advisor for AbbVie Inc., a speaker for Teva and has received research support from AbbVie Inc., Biotie, the National Institutes of Health, and the Veterans Administration. C.H., K.C., S.E., and J.A.B. are employees of AbbVie Inc. and hold stock or stock options.

## Supporting information

Additional Supporting Information may be found in the online version of this article at the publisher's web‐site.

Supplementary Information Figure 1

Supplementary Information Table 1

## References

[1] Nussbaum RL , Ellis CE . Alzheimer's disease and Parkinson's disease. N Engl J Med 2003;348(14):1356‐1364. 12672864 10.1056/NEJM2003ra020003

[2] Hornykiewicz O . Basic research on dopamine in Parkinson's disease and the discovery of the nigrostriatal dopamine pathway: the view of an eyewitness. Neurodegener Dis 2008;5(3‐4):114‐117. 18322366 10.1159/000113678

[3] Agid Y , Ahlskog E , Albanese A , et al. Levodopa in the treatment of Parkinson's disease: a consensus meeting. Mov Disord 1999;14(6):911‐913. 10584663 10.1002/1531-8257(199911)14:6<911::aid-mds1001>3.0.co;2-h

[4] Nutt JG . Pharmacokinetics and pharmacodynamics of levodopa. Mov Disord 2008;23(suppl 3):S580‐S584. 18781675 10.1002/mds.22037

[5] Shoulson I , Glaubiger GA , Chase TN . On‐off response. Clinical and biochemical correlations during oral and intravenous levodopa administration in parkinsonian patients. Neurology 1975;25(12):1144‐1148. 812004 10.1212/wnl.25.12.1144

[6] Poewe W , Antonini A . Novel formulations and modes of delivery of levodopa. Mov Disord 2015;30(1):114‐120. 25476691 10.1002/mds.26078

[7] Sharma S , Singh S , Sharma V , Singh VP , Deshmukh R . Neurobiology of l‐DOPA induced dyskinesia and the novel therapeutic strategies. Biomed Pharmacother 2015;70:283‐293. 25776513 10.1016/j.biopha.2015.01.029

[8] Hechtner MC , Vogt T , Zollner Y , et al. Quality of life in Parkinson's disease patients with motor fluctuations and dyskinesias in five European countries. Parkinsonism Relat Disord 2014;20(9):969‐974. 24953743 10.1016/j.parkreldis.2014.06.001

[9] Pahwa R , Tanner CM , Hauser RA , et al. Amantadine extended release for levodopa‐induced dyskinesia in Parkinson's disease (EASED Study). Mov Disord 2015;30(6):788‐795. 25650051 10.1002/mds.26159 PMC5024015

[10] Katzenschlager R , Hughes A , Evans A , et al. Continuous subcutaneous apomorphine therapy improves dyskinesias in Parkinson's disease: a prospective study using single‐dose challenges. Mov Disord 2005;20(2):151‐157. 15390035 10.1002/mds.20276

[11] Pouratian N , Thakkar S , Kim W , Bronstein JM . Deep brain stimulation for the treatment of Parkinson's disease: efficacy and safety. Degener Neurol Neuromuscul Dis 2012;2012(2). doi: 10.2147/DNND.S25750 10.2147/DNND.S25750 PMC3843969 24298202

[12] Olanow CW , Kieburtz K , Odin P , et al. Continuous intrajejunal infusion of levodopa‐carbidopa intestinal gel for patients with advanced Parkinson's disease: a randomised, controlled, double‐blind, double‐dummy study. Lancet Neurol 2014;13(2):141‐149. 24361112 10.1016/S1474-4422(13)70293-X PMC4643396

[13] Fernandez HH , Standaert DG , Hauser RA , et al. Levodopa‐carbidopa intestinal gel in advanced Parkinson's disease: final 12‐month, open‐label results. Mov Disord 2015;30(4):500‐509. 25545465 10.1002/mds.26123 PMC4674978

[14] Slevin JT , Fernandez HH , Zadikoff C , et al. Long‐term safety and maintenance of efficacy of levodopa‐carbidopa intestinal gel: an open‐label extension of the double‐blind pivotal study in advanced Parkinson's disease patients. J Parkinsons Dis 2014;5(1):165‐174. 10.3233/JPD-140456 25588353

[15] Hauser RA , Friedlander J , Zesiewicz TA , et al. A home diary to assess functional status in patients with Parkinson's disease with motor fluctuations and dyskinesia. Clin Neuropharmacol 2000;23(2):75‐81. 10803796 10.1097/00002826-200003000-00003

[16] Olanow CW , Obeso JA , Stocchi F . Continuous dopamine‐receptor treatment of Parkinson's disease: scientific rationale and clinical implications. Lancet Neurol 2006;5(8):677‐687. 16857573 10.1016/S1474-4422(06)70521-X

[17] Adamiak U , Kaldonska M , Klodowska‐Duda G , et al. Pharmacokinetic‐pharmacodynamic modeling of levodopa in patients with advanced Parkinson disease. Clin Neuropharmacol 2010;33(3):135‐141. 20216409 10.1097/WNF.0b013e3181d47849

[18] Iravani MM , Jenner P . Mechanisms underlying the onset and expression of levodopa‐induced dyskinesia and their pharmacological manipulation. J Neural Transm 2011;118(12):1661‐1690. 21881839 10.1007/s00702-011-0698-2

[19] Olanow CW , Gauger LL , Cedarbaum JM . Temporal relationships between plasma and cerebrospinal fluid pharmacokinetics of levodopa and clinical effect in Parkinson's disease. Ann Neurol 1991;29(5):556‐559. 1859185 10.1002/ana.410290516

[20] Tel BC , Zeng BY , Cannizzaro C , Pearce RK , Rose S , Jenner P . Alterations in striatal neuropeptide mRNA produced by repeated administration of L‐DOPA, ropinirole or bromocriptine correlate with dyskinesia induction in MPTP‐treated common marmosets. Neuroscience 2002;115(4):1047‐1058. 12453478 10.1016/s0306-4522(02)00535-3

[21] Contin M , Martinelli P . Pharmacokinetics of levodopa. J Neurol 2010;257(suppl 2):S253‐S261. 21080186 10.1007/s00415-010-5728-8

[22] Lang AE , Widner H . Deep brain stimulation for Parkinson's disease: patient selection and evaluation. Mov Disord 2002;17(suppl 3):S94‐S101. 11948762 10.1002/mds.10149

[23] Weaver FM , Follett K , Stern M , et al. Bilateral deep brain stimulation vs best medical therapy for patients with advanced Parkinson disease: a randomized controlled trial. JAMA 2009;301(1):63‐73. 19126811 10.1001/jama.2008.929 PMC2814800

[24] Volkmann J , Albanese A , Antonini A , et al. Selecting deep brain stimulation or infusion therapies in advanced Parkinson's disease: an evidence‐based review. J Neurol 2013;260(11):2701‐2714. 23287972 10.1007/s00415-012-6798-6 PMC3825542

[25] Stocchi F , Rascol O , Kieburtz K , et al. Initiating levodopa/carbidopa therapy with and without entacapone in early Parkinson disease: the STRIDE‐PD study. Ann Neurol 2010;68(1):18‐27. 20582993 10.1002/ana.22060

[26] Pilleri M , Antonini A . Therapeutic strategies to prevent and manage dyskinesias in Parkinson's disease. Expert Opin Drug Saf 2015;14(2):281‐294. 25483147 10.1517/14740338.2015.988137

