# Supplementary figures and images for: Effect of levodopa‐carbidopa intestinal gel on dyskinesia in advanced Parkinson's disease patients
Source: Mov Disord. 2016 Jan 28;31(4):530–7. doi: 10.1002/mds.26528 (PMC5066747; doi:10.1002/mds.26528)

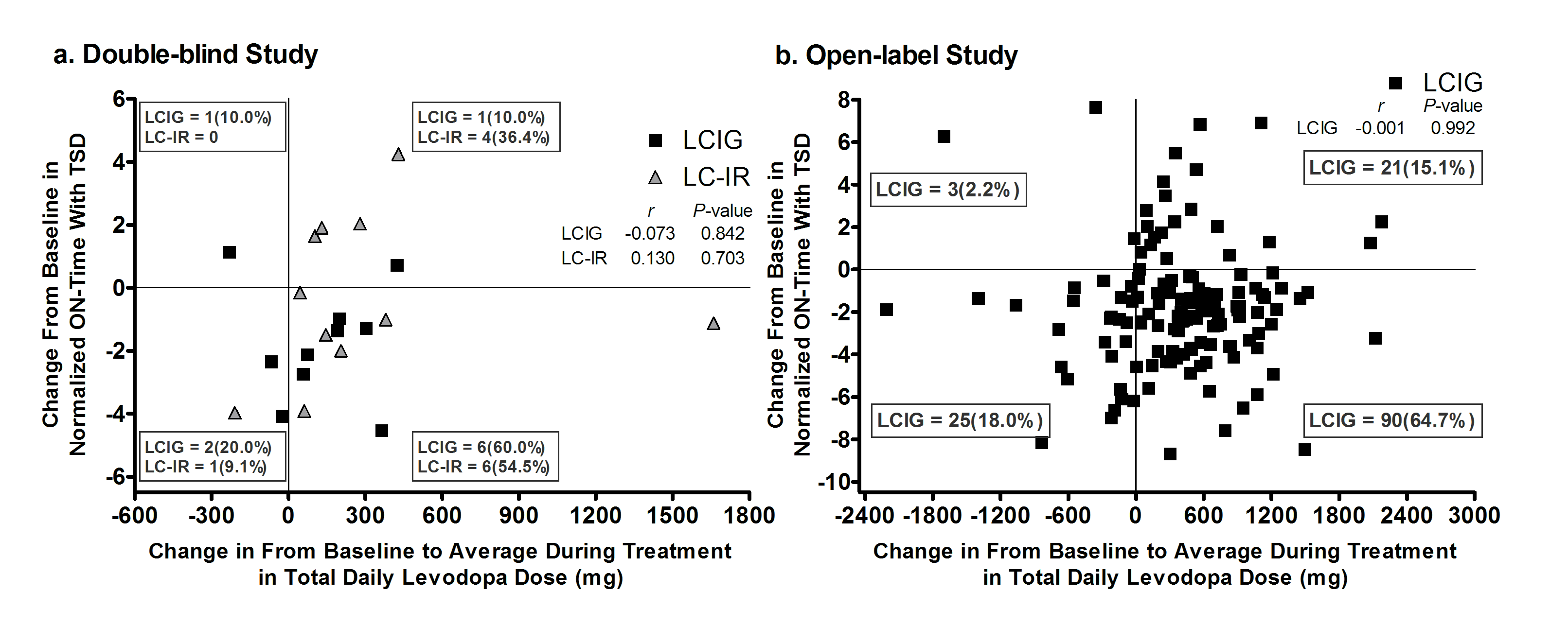

Supplement: Supplementary file 1 — Supplementary Information Figure 1 [file MDS-31-530-s001.tif]
