# Supplementary material for: Effect of levodopa‐carbidopa intestinal gel on dyskinesia in advanced Parkinson's disease patients
Source: Mov Disord. 2016 Jan 28;31(4):530–7. doi: 10.1002/mds.26528 (PMC5066747; doi:10.1002/mds.26528)
Supplement: Supplementary file 2 — Supplementary Information Table 1 [file MDS-31-530-s002.docx]

**Supplemental Table 1. Mean Change from Baseline to Final in “On” Time with Troublesome Dyskinesia in Subgroups of Patients from the Open-label Study with Elevated Dyskinesia at Baseline**

| Subgroup Based on Mean Hours of TSD At Baseline |  | Hours of “On” Time with TSD | |
| --- | --- | --- | --- |
|  | n | Mean (SD)  At Baseline | Mean (SD) Change from Baseline to Final |
| All Patients | 306 | 1.60 (2.03) | -0.36 (2.77) |
| ≥ 1 hour | 139 | 3.37 (1.81) | -1.83 (2.92) |
| ≥ 1.5 hours | 118 | 3.74 (1.72) | -2.22 (2.87) |
| ≥ 2 hours | 101 | 4.08 (1.63) | -2.52 (2.80) |
| ≥ 2.5 hours | 84 | 4.44 (1.56) | -2.76 (2.93) |
| ≥ 3 hours | 71 | 4.77 (1.47) | -3.08 (2.98) |

TSD = troublesome dyskinesia; SD = standard deviation
